# Diseases of the Rectum

**Published:** 1838-01-01

**Authors:** 


### Medico-Chirurgical Review,

### No. LV.

**[No. 15 of a Decennial Series.]**

OCTOBER 1, 1837, TO JANUARY 1, 1838.

### DISEASES OF THE RECTUM.

I. A Treatise on the Malformations, Injuries, and Diseases of the Rectum and Anus. Illustrated with Plates. By *George Bushe*, M.D., formerly Professor of Anatomy and Physiology, &c. 8vo., pp. 299. New York, 1837.

II. On the Diseases of the Rectum. By *James Syme*, F.R.S.E., Professor of Clinical Surgery in the University of Edinburgh. 8vo., pp. 138. Edinburgh, 1838.

The diseases of the rectum are so frequent in all classes—so troublesome when they occur—and, from the seat of the disorder, as well as from its nature, so much an object of annoyance to the patient—that an accurate acquaintance with them is absolutely indispensable on the part of the practical surgeon.

It is an object of general remark, we may add of general regret, that the works which we possess on this particular subject are far from having obtained or merited a high character. They would appear, for the most part, to have emanated from gentlemen anxious for experience, rather than from those who are possessed of it—from gentlemen whose books were intended to become the parents of patients, instead of patients having been the parents of the books. The information which such works can give may be guessed with little difficulty. Excellent handbills, they form indifferent manuals; and the reader rises from their careful study a more accomplished advertiser, but probably not a better surgeon. The long series of books of this description have bred a species of despair, and the idea has taken root, that, beyond the treatment of fistula and piles, the surgery of the rectum is a sort of land of the Cimmerians, where quacks alone can breathe, and humbug darkens the air.

Perhaps this is an idle fancy and an injurious imputation; but facts would appear to have given some colour to it—facts would seem to shew, that when once a man plunges into the rectum, he leaves decency behind, and, in the midst of fæces, his practices grow foul and his habits nasty. "In for a penny, in for a pound," suggests itself as a natural maxim, and, being in a hole which daylight never enters, his arts are shrouded in darkness and impunity. More than one person has made a fortune by curing strictures which never existed, and passing bougies where they never were wanted. On the "blind side" of his patient in every way, he has readily persuaded him of the truth of what his own fears suggested; and after years of suspended cure, but not suspended payment, he has allowed him to escape with a rectum no better (fortunate if no worse), a constitution damaged, and a purse lightened. Bath, perhaps, may have contained such cases and such surgeons; and if the quack has been at length exposed, it is only after the work has been done—it is only after the patient has suffered, and the doctor has grown rich.

No successful attempts have yet been made to apply to the diseases of the rectum the pathological knowledge of the day; yet, no doubt, they are as susceptible as other maladies of exact observation, of scientific analysis, and of simple and appropriate treatment. It is not that new diseases are either to be discovered or described—it is not that new remedies or monstrous operations are to be invented or suggested—but it is that the diseases which exist, and the remedies which are more or less familiarly known, should be properly associated and distinguished, and that the vagueness, disputes, and mystery which now obtain should be dispelled. The application of what we know of the affections and dispositions of the tissues has not yet been properly made in this instance.

The authors of the works before us, Mr. Syme and Dr. Bushe, write from very different, we might say from opposite, quarters—Mr. Syme from Edinburgh, Dr. Bushe from New York. We shall presently see in what they disagree, and in what they coincide—what opportunities of observation they have enjoyed, and what varieties of treatment they propose.

We intend to devote more than one article to the subject of diseases of the rectum. We will take the more common affections first, and afterwards consider the unusual and the complicated. We purpose comparing on each subject the two authors, and taking for our text whichever is the more accurate or the more luminous.

The object of each author may, perhaps, be best displayed by his own words, in his own preface. Mr. Syme informs us that—

"The diseases of the rectum are very frequent in their occurrence, and derive additional interest from the distressing symptoms which they occasion, as well as the relief of which they admit from the resources of surgical art. It may be added, that the mystery and concealment connected with their situation not only favour the deceptions of empirical practitioners, but also encourage the proceedings of wrong-headed operators, who prefer the most painful and dangerous means of treatment to those which are easy and safe.

On these accounts, it is desirable that this department of surgery should be thoroughly understood by the members of the profession, and that its leading principles should be placed prominently before them. The diseases of the rectum have accordingly been made the subject of many treatises expressly devoted to their consideration, and it may seem unnecessary for me to increase the number of these productions. But the progress of modern pathology and surgical practice has introduced many improvements that have not yet been fairly brought together, and explained in their application to the management of those complaints which are at present more particularly in view. I have attempted to supply this defect; and, by a plain statement of the seat, nature, symptoms, and treatment of the different affections which are met with at the extremity of the rectum, endeavoured to assist practitioners in discharging their duty to the patient, and to protect patients against unprincipled or reckless practitioners."

For pretty nearly the same reasons Bushe was induced to study and to publish.

"Many years ago," he says, "I was induced to pay particular attention to the diseases of the rectum and anus, in consequence of their frequency, and the diversity of opinion which prevailed in relation to their nature and treatment. My opportunities for investigating them have been ample, and I may safely say, that I spared neither time, trouble, nor expense, in endeavouring to arrive at just conclusions. In the compilation of my researches, I have aimed at simplicity and conciseness, at the same time that I have been careful to omit nothing of importance."—*Advert.*

Dr. Bushe goes on to lament the imperfections which he would willingly have seen removed from his pages. But ill-health prevented his giving effect to his wishes, and we regret to say that since that apology was penned, the death of Dr. Bushe has but too decisively established its sincerity.

Dr. Bushe's work is more comprehensive in its scope than Mr. Syme's. It treats in the following order of the following subjects: The Anatomy of the Rectum—Functions of the Rectum—Malformations of the Rectum and Anus—Foreign Bodies in the Rectum—Laceration of the Rectum—Inflammation of the Rectum—Inflammation and Excoriation of the Anus—Inflammation of the Rectum and Anus, arising from the application of Gonorrhœal Matter—Fissure of the Anus—Neuralgia of the Extremity of the Rectum—Spasmodic Contraction of the Sphincter Ani—Ulceration of the Rectum—Venereal Ulceration of the Rectum—Affections called Hemorrhoidal—Enlargement of the Hemorrhoidal Veins—Prolapsus of the Rectum—Relaxation of the Anus—Relaxation of the Rectum, with Invagination of the Mucous Membrane—Itching of the Anus—Excrescences about the Anus—Polypi of the Rectum—Abscess near the Anus—Fistula in Ano—Contraction of the Anus—Stricture of the Rectum—Carcinomatous Degeneration of the Rectum.

Mr. Syme's book contains six chapters. The first is on Fistula in Ano—the second on Hæmorrhoids—the third on Prolapsus Ani—the fourth on Polypus of the Rectum—the fifth on Stricture of the Rectum—the sixth on Spasmodic Stricture of the Rectum.

We do not think it necessary to say anything at present on the anatomy or functions of the rectum. We shall therefore pass to affections not extremely rare, and of the utmost consequence—affections for which the surgeon is obliged to act with promptitude, and frequently with risk.

### I. Malformations of the Rectum and Anus.

We now devote ourselves exclusively to Dr. Bushe. His arrangement of the malformations of the rectum and anus appears to be both simple and complete, at all events, for practical purposes. It is as follows:—

Imperforation of the anus. { Incomplete.
Complete.

Imperforation of the Rectum. { By one partition.
By two partitions.
By puckering and induration of its walls.

| | |
|---|---|
| Unnatural termination of the Rectum. | In the bladder or urethra.<br>In the vagina.<br>In the sacral region.<br>In two extremities.<br>In a cloaca with the vagina and urethra. |
| Termination of other organs in the rectum | Of the ureters.<br>Of the vagina. |
| Absence of the rectum. | |

A. *Imperforation of the Anus.*

When *incomplete,* this consists either in an extension of skin over the border of the sphincter ani, or in a contraction of the extremity of the rectum.

The *complete* is a much more common form of anomaly than that which has just been described, and is produced by a lamina of fibro-cellular tissue, surrounded by more or less puckering of the adjoining skin. In most cases this membrane is so thin and transparent, that when the infant strains, the meconium forces it downward, thus constituting a dusky, fluctuating tumor. However, in some rare instances, it is thick, hard and unyielding, particularly at the circumference ; for, in the centre, it is almost invariably so thin, that the meconium can be seen through it.

B. *Imperforation of the Rectum.*

*By one partition.* The situation of this, says Dr. Bushe, varies from two lines, to an inch or more above the anus. In the majority of cases, it is thin, and transparent, though occasionally thick and hard. The anus is always well formed, but we are soon apprised of the nature of the case, by the retention of the meconium, by the inability of the nurse to throw up injections, and by examination with the extremity of the little finger.

By *two partitions.* Their site as well as structure differ. In a new-born child brought into the dissecting-room, the upper part of the rectum was loaded with meconium, the partitions were thin and friable, being about three-quarters of an inch apart, while the lowermost was nearly half an inch from the anus.

*By puckering and induration of its walls.* The only case Dr. Bushe has ever read of, occurred to Engerran. In this instance, so great was the induration and puckering, that it presented the appearance of a knob, or knot in the intestine.

C. *Unnatural Terminations of the Rectum.*

*In the bladder or urethra.* " When the rectum terminates in the urinary organs, it opens either obliquely between the ureters into the neck of the bladder, or into the posterior part of the urethra. It generally tapers down very considerably before it arrives at its destination ; though, in some few instances, (one of which I have seen,) it terminates *cæco fine,* about half an inch above which a narrow tube passes off anteriorly to communicate with the bladder or urethra. In either case the recto-vesical orifice is so small, that only the thinner part of the meconium can be evacuated ; and thus it is, that the unfortunate infant generally dies within a week from its birth.

When the opening is vesical, the meconium and the urine are mixed ; but, when urethral, a small jet of meconium generally precedes the passage of the urine.

This malformation is much more common in males, and from the length, narrowness, and curvature of the urethra, is much more dangerous than in females. It is often accompanied with imperfect development of the genito-urinary organs, especially, with imperforation of prepuce and urethra.

Though rarely, the anus sometimes exists in these cases, and permits the entrance of a probe for a few lines." 42.

*In the vagina.* The rectum may open into any portion of the posterior or lateral walls of the vagina. The orifice in this case is much larger than in the last malformation, which, together with the greater width and straightness of the vagina, renders it far less fatal. However, the mucous membrane becomes more or less excoriated, ulcerated, indurated and fungated, in almost every case, and abscesses form in the adjacent cellular tissue. As in the last lusus, there may be an external opening in the natural state of the anus.*

*In the sacral region.* A portion of the sacrum may be so deficient as to permit the extremity of the rectum to pass through it, and open externally. Dr. Bushe only knows of one case, recorded by La Faye, in the Principes de Chirurgie.

*In two extremities.* Dr. Bushe examined a healthy child, a few days old, in whom the rectum terminated by two extremities, one being placed a little more anterior than natural, while the other, though also on the median line, was situated nearly an inch further back. This last, which was the smaller of the two, did not discharge more than one-third of the fæces, and appeared to be about an inch and a half in length.

"D. *Termination of other Organs in the Rectum.*

*Of the ureters.* Cases of this kind are exceedingly rare, and when they do occur, the ureters generally enter a short distance below the line of reflection of the peritoneum. Most commonly other malformations exist at the same time; among which, absence of the urethra in the female is the most frequent.

*Of the vagina.* This is also a very uncommon anomaly. When present, the urethra generally occupies its natural situation; the menstrual discharge issues from the anus, and impregnation, nay even parturition, has been safely effected in such cases, through this orifice, which will be enlarged by more or less laceration of the perineum.

*In a cloaca with the urethra, and vagina.* It may so happen that the vagina, urethra and rectum, terminate together in the perineum; thus constituting a species of common vestibule or cloaca, similar to that of the annotrêmes, and of a great number of other animals. Saviard observed an anomaly of this kind, in a new-born infant, which, as far as I know, is the only one on record." 45.

E. *Absence of the Rectum.*

The absence of a portion is much more common than that of the entire rectum; which, in such cases terminates in a cul-de-sac at a grearter or less distance from the surface.

When this intestine is completely wanting, the extremity of the colon either floats in the abdominal cavity, hangs into the pelvis, or is bound

* We may observe that in the description of these malformations we are obliged to employ the ipsissima verba of the author. Abbreviation is impossible. But we omit the bibliographical notes.

down to the top of the sacrum. In some instances a fleshy, ligamentous, or fatty cord is attached to the cul-de-sac of the colon, or portion of the rectum, that may be present, and passes downwards, following the direction of the sacrum, to be blended, in many instances, with the cellular tissue behind the prostate and neck of the bladder.

"A preternatural anus may exist, and then it will occupy some situation in the face, neck, thorax, or abdomen."* 46.

When the rectum is either partially or totally wanting, the pelvis is generally contracted.

In some instances, the anus is well formed, and permits the entrance of a sound for a few lines ; but generally, there is no trace of this opening, the skin being thick, hard, and, in the majority of cases, supported by muscle.

From the retention of the contents of the intestines caused by any of the malformations now mentioned, there arises great pain, as manifested by pitiful cries—abdominal enlargement with tension and shining of the integuments—discolouration and swelling of the face—inflation of the scrotum and penis—difficult and irregular respiration, caused partly by the pain resulting from the descent of the diaphragm on the inflamed intestines, and partly by the increased size of the abdomen—frequency, smallness and irregularity of the pulse—vomiting—straining to stool—hiccup—coldness with flexion of the extremities, and convulsions.

In some instances the colon bursts, and its contents are poured into the peritoneal cavity.

The majority of these malformations are fatal. When there is no outlet, death soon occurs; but in those instances in which there exists a small opening, even into another organ, this event takes place more slowly. On dissection, the intestines are found distended with gas and fæces, and are highly inflamed.

### TREATMENT.

For some of the simpler of the preceding malformations, surgery offers not only alleviation but a cure; but it must be confessed that it presents but doubtful chances of recovery from the more severe. Still, in the worst cases, operations have every now and then been fortunate, and the surgeon should be acquainted with those glimmerings of success which twinkle amidst the mass of failures.

* "Mery mentions a case in which the colon opened at the umbilicus, the rectum being absent. (Hist. de l'Acad. des Scienc. p. 40. 1700.) Littre records another, in which the ilium terminated above the pubis. (Mem. de l'Acad. des Scienc. p. 9. 1709.) Petit has described a case in which the ilium opened at the left side of the bas-ventre, and thus formed an anus. (Ibid. p. 89. 1716.) Dinmore mentions a remarkable case of an infant in whom the inferior portion of the abdomen was badly developed, while the intestine turned upwards, and opened under the border of the right scapula. A still more extraordinary case is related by Bils, in which the intestine mounted from the pelvis, through the chest, into the neck, and opened on the face by a very small orifice. (Specimen Anat. Rotterdam. p. 10. 1661.)

### A. *Incomplete Imperforation of the Anus.*

When this is caused by a prolongation of skin, the superabundant integument ought to be divided in two or more points, and meshes of lint, or gum-elastic bougies, besmeared with simple ointment, then introduced, renewed daily, increased successively in volume, and continued for months. If, however, the extremity of the intestine be merely contracted, it will seldom be necessary to have recourse to the knife.

### B. *Complete Imperforation of the Anus.*

This is very curable too. The membrane which shuts up the extremity of the intestine, should be divided crucially with a sharp-pointed straight bistoury, and the angles of the flaps thus formed, removed with a forceps and curved scissors. The means adopted for keeping the aperture patent should be the same as have been already mentioned.

When the membrane covering the extremity of the intestine is prolonged forward to the perineum, where is a small hole through which the thinner part of the fæces drain off, it should be first divided from before backwards, with a probe-pointed bistoury introduced into the foramen, and the rest of the operation then conducted as above described.

### C. *Imperforation of the Rectum.*

If this is produced by a fine membrane, we may be able to break it down with the extremity of the little finger; but if it be hard, we ought, provided we feel the fluctuation of meconium, to pierce it with a trochar, and then dilate the opening thus formed, with sponge-tents, &c.

### D. *Termination of the Rectum in the Bladder or Urethra.*

If there are urgent symptoms, the surgeon must dissect for the extremity of the intestine. It has been advised to open the neck of the bladder, but this only evacuates the bladder itself. If the bowel opens into the urethra, and the opening is divided in passing the knife into the bladder, the operation will prove unavailing, first, because the division must be in the direction of the rectal canal, secondly, because it must be limited to a small extent, and thirdly, because in these cases, as before-mentioned, the rectum becomes narrow before it enters into the bladder or urethra.

### E. *Termination of the Rectum in the Vagina or Vulva.*

In either of these cases, Dr. Bushe is of opinion, and we agree with him, that if the fæces can be easily discharged, surgical interference is improper. But there have been several opinions to the contrary. Thus, when the opening is vaginal, Vicq. d'Azyr recommended the division of the posterior wall of the vagina, below the opening, and also as much of the subjacent tissues as would admit the introduction of a canula. Martin improved upon this operation in advising the flaps of the vagina to be united in front of the canula by points of suture; and more recently Velpeau has proposed facilitating its performance, first, by introducing a blunt hook into the *cul-de-sac* of the rectum, and then rendering its extremity prominent in the perineum, secondly, by dividing the parts covering the extremity of the hook, and thirdly, by passing the tube into the opening thus formed. If we perform this operation, Velpeau's method is certainly preferable, when practicable; which can only be when a *cul-de-sac* exists; for the intestine tapers down very considerably in some cases before it enters. Breschet has proposed the same operation as Vicq. d'Azyr, when the opening is in the *fourchette.*

F. When the rectum opens through the sacrum—when it bifurcates—or, when there is a common opening for the urethra, vagina, and rectum, surgery can offer no assistance.

G. *Partial absence of the Rectum.*—When this is the case, which cannot be told à priori, an anus ought to be made, if possible, in the natural situation. The following are Dr. Bushe's directions:

" The little patient being held in the lap of an assistant, with the knees bent and the buttocks thrown forward, the surgeon should make an incision of about eight or ten lines, from before backwards, in the accustomed situation of the anus. If, in the course of the dissection, he discovers the sphincter or the levatores ani muscles, he should separate their fibres carefully, and prosecute the dissection nearly in the axis of the body, or almost perpendicularly, cutting from before backwards, to avoid wounding the bladder; at the same time he should be careful not to get behind the rectum,—a mistake which has sometimes occurred during the dissection. The blood ought to be well sponged out, and the fore-finger of the left hand repeatedly used to seek for the rectum. If after dissecting two inches, or at most two and a half inches deep, the intestine cannot be detected, the operation ought to be abandoned; but, if the bowel be discovered by its blackness and fluctuation, either a trochar, or, what is better, a bistoury, should be forced into it, and the meconium evacuated. The opening thus formed should be maintained by tents of prepared sponge, meshes of lint, besmeared with cerate, or gum elastic tubes, kept continually introduced. The operation ought to be conducted with as much despatch as is compatible with safety, for pain never fails to prostrate babes. Most surgeons who have performed such operations have been unsuccessful; thus Petit mentions three fatal cases, death having occurred within a few hours, in one from convulsions, and in another from exhaustion. However, other surgeons have succeeded; among whom we may mention, Roux-Brignoles, and Sanson.

Should the little patient recover from the operation, his comfort will afterwards depend, first, upon there being a sphincter, and the opening having been made through it, and secondly, upon the proximity of the rectum to the skin. If there be no sphincter, he must be miserable indeed; and should the space between the rectum and the skin be great, he will labour under an affliction not to be endured." 53.

H. It is only *when the rectum cannot be found,* that there is any pretext for performing an operation, first proposed by Littre—that of opening the sigmoid flexure of the colon. If, after a careful dissection in the perineum, the rectum cannot be discovered, there seems no alternative between leaving the child to its fate, and opening the colon by an operation. Suppose the latter decided on, the steps to be taken are these:—

" The infant being extended on a soft pillow, an incision of the skin, subjacent, cellular tissue, and fascia, should be made from one to two inches in length, between the anterior and superior spinous processes of the ilium, and the pubes, situated a little above Poupart's ligament. The different layers of the abdominal parietes ought then to be divided successively with a bistoury on a director, until the operator arrives at the peritoneum, which should be pinched up, and divided to the same extent as the external parts. By this last section, the intestine will be exposed, bearing a dark colour, and greatly distended with meconium. The left fore-finger ought now to be introduced so as to bring the bowel as far outward as possible, while with the right hand, a large, soft ligature, should be carried through its mesentery by means of a curved silver needle. This being accomplished, a longitudinal incision should be made into the intestine, and the meconium evacuated ; after which a mesh of anointed lint ought to be carefully introduced, so as to prevent the adhesion of the lips of the wound. Finally, each end of the ligature should be so secured, on either side of the abdominal wounds, as to maintain the parietal and intestinal openings *in situ*. If the child survives, the ligature may be removed on the fourth day, as by that time the intestine will be firmly consolidated with the wound of the abdominal parietes.*

Callisen has, in preference to the operation of Littre, proposed opening the cæcum, or descending colon in the lumbar region, by an incision in the course of the border of the quadratus lumborum muscle, by which the peritoneum is left untouched. This operation has never been performed but once, then by Roux, and in this case the child died in two hours.

Dubois first gave the idea, afterwards carried into effect by Martin, of opening the sigmoid flexure of the colon, and then passing a sound through it towards the perineum, so as, if possible, to render it salient, and thus create a certain mark for our incisions in the perineum. It is to be hoped that no surgeon at the present day would be so cruel, as to subject an infant to this double operation, however ingenious and imposing it may appear at first sight." 56.

This closes the account of the malformations of the rectum. It is by no means a bad one—indeed, for practical purposes, it is a very succinct, and yet sufficiently complete one. These malformations are by no means a matter of mere curiosity. Within these ten years we have ourselves seen six cases, all requiring operations. When they occur, no time is to be lost, and the surgeon's ignorance or knowledge soon becomes apparent. It is therefore worth every practitioner's while to be well acquainted with these observations from the natural disposition of the bowel, and to be well acquainted also with the surgical measures, which experience has decided to be best adapted for remedying each particular evil. This consideration has induced us to devote so many pages to the subject. We now quit it.

### II. Foreign Bodies in the Rectum.

These form the contents of the fourth chapter of the work of Dr. Bushe, and are not treated of at all in that of Mr. Syme. We, therefore, still cleave exclusively to the former.

The foreign bodies frequently found in the rectum may be divided, says Dr. Bushe, into two classes, viz.: Those which are generated in consequence

---

* "Dessault performed this operation on a child 48 hours after birth, but it was fatal. (Journal de Chirurg. tome iv. p. 248.) In 1783, Dubois operated on a child three days old, death however occurred ten days afterwards. (Recueil Periodique de la Societé de Med. tome iii. p. 125.) Duret, a naval surgeon at Brest, successfully operated on a child three days old. In this case the vomiting ceased immediately ; the thread supporting the bowel was removed on the fifth day ; on the sixth the opening was partially closed by the extrusion of the bowel, and on the seventh the infant was cured. (Ibid. tome iv. p. 45.)"

of diseased action of the digestive organs, and those which are either swallowed or introduced through the anus. The former embrace biliary, intestinal and fæcal concretions; while the latter include pins, nails, fruit-stones, coins, small bones, &c., taken in by the mouth; or pieces of wood, cork, meat, bone, horn, ivory and metal, pots, cups, bottles, ferrules, rings and the like, forced into the anus, either completely or incompletely, sometimes by the individual himself, with a view to obviate costiveness, or in consequence of a perverted imagination; but more commonly by wicked persons, who generally take advantage of the inebriated state of their intended victim.

Unless the foreign body be either very large, or of such a form as to interfere with its exit, it will be discharged along with the fæces. If it is not, irritation, prolapsus of the mucous membrane, and inflammation, first in the part, then in neighbouring parts, and lastly in the bowels generally, will be the consequences.

The removal of such bodies must be effected by the surgeon. Various instruments will be required, according to the circumstances of the case. In the different instances on record, some of the more curious or instructive of which we shall quote, most of the following modes or instruments have been employed: blunt hooks of different sizes and shapes, a lever, gimlet, cutting forceps, strong long scissors with probe points, a six-inch narrow saw, wooden gorgeret, polypus and lithotomy forceps of different shapes and sizes, a speculum, strong waxed ligatures, metallic tubes of various length and size, and a probe-pointed bistoury; to all of which the crooked finger and a small hand have proved admirable adjuncts.

In particular circumstances, particular precautions or manipulations are requisite.

A. *When the foreign body is large or spiculated*, it may be necessary to divide the sphincter. Mr. Hevin records a case, which was related to him by M. Tostain, a surgeon of Saint Lo, in which a bone that had been arrested in the œsophagus was forced by a probang into the stomach. While there it caused great pain, and also in its course through the intestines, which occupied about a month. The patient, who had for years been subject to hemorrhoids, at this time found the irritation at the anus much increased; he therefore consulted Tostain, who, in place of hemorrhoids, found a bone, one extremity of which had pierced the rectum, the flesh, and even the skin, the body being within the intestine, while some other points were engaged in the mucous membrane. To extract it, Tostain was obliged to make a small incision in the walls of the rectum. All the bad symptoms subsided, and the patient was well in eighteen days.

But the anus is very dilatable, and incisions of the sphincter may frequently be dispensed with. Dr. Bushe relates an interesting case in proof of this. He was consulted in the case of a delicate female, 35 years of age, who for seven years had been subject to constipation and repeated attacks of colic; the former had increased, attended with sickness of stomach, while the latter became more frequent, and from which she onlv experienced relief when her bowels were moved,—a task not accomplished without the most painful efforts and very great difficulty, much cathartic medicine and powerful enemata being necessary in each succeeding attack.

"I was called to visit her in one of those paroxysms, and found her sallow, emaciated and dejected. From the severe bearing down pains, together with the sense of weight and fulness in the sacral region, which she complained of, I was led to make an examination of the rectum, when I found the mucous membrane slightly protruding from the anus, and very turgid, the sphincter excessively irritable, and a large concretion distending the pouch of the rectum. I now apprized her of the nature of the case, and the absolute necessity of removing the foreign body, to which she willingly consented. Having placed her hips over the edge of the bed, and bent her knees towards her chin, while she lay on her back, I introduced a strong and long lithotomy forceps, with which cautiously laying hold of the concretion, I slowly and steadily extracted it, with no more injury than slight laceration of the mucous membrane; although on measurement it proved to be six inches and three quarters in circumference, and two inches and a half in length. The bowels were then freely evacuated by injections; leeches and fomentations were applied to the anus, the recumbent position was enjoined, and a speedy recovery ensued." 61.

B. *If the foreign body is of inflexible solid or vegetable substance*, it may be found advantageous to fix it with the fore-finger of the left hand, while we bore a hole in and extract it with a gimlet. Thus Saucerotte withdrew a piece of wood, three inches in length and two in width, with a cork-screw, which he inserted into the wood, while he steadied it with the fore-finger of his left hand. Bruchman performed a similar operation with a gimlet.

C. *If the foreign body is hollow or brittle*, an intelligent boy can best withdraw it by the hand well oiled and cautiously introduced into the rectum. In a note, Dr. Bushe quotes a curious case related by Nole, surgeon to the King of France and Marine Hospital at Brest.

*Case.* A monk wishing to get rid of a violent colic, introduced into the rectum a bottle of Hungary water, (these bottles are generally long,) through the cork of which he had made a small opening to permit the fluid to flow into the intestine. In his anxiety to perform the operation well, he pushed the bottle so far that it completely entered into the gut. He could neither go to stool nor receive a lavement. A *sage femme* failed to insert her hand; the forceps and speculum were tried in vain; however, a boy, from eight to nine years of age, succeeded in introducing his hand, and removed the bottle.

Dessault, in endeavouring to extract a porcelain jelly pot by means of forceps, broke it; yet he got out the fragments.

D. *When a piece of bone, ivory, wood, or horn, is fixed across the intestine*, it may be extracted with the forceps, finger, or hand.

The following case is related by Hevin.

*Case.* M. Quesnay pushed a bone, which was arrested in the œsophagus, into the stomach. Afterwards this body presented itself near the orifice of the rectum. The patient, tormented with pain, called on Quesnay, who introduced his finger into the anus, and found the bone placed obliquely across the gut, with its inferior extremity fixed into its walls. He passed a forceps along his finger, and having seized the bone superiorly, lifted it up, thus disengaging its inferior portion. He then grasped it lower down, and removed it without difficulty or pain.

Another case, detailed by M. Thiandiére, is curious.

*Case.* " A man, aged twenty-two, who, with a view to overcome costiveness, introduced a forked stick into the rectum. This stick was five inches long; one prong was an inch and a half longer than the other, and they were separated to the extent of two inches, each prong being about four lines in diameter, and the stem formed by their union half an inch. He inserted the stem first, and when the short prong had entered the bowel, he endeavoured, by dragging on the long one, to force out the indurated feces. In this ingenious essay it is unnecessary to say that he failed completely; the pain being very severe, he ceased his manipulations, and finding it impossible to withdraw the fork, he forced the long prong completely within the anus, with the extraordinary idea that it would be consumed with the food. Fearful to divulge the nature of his case, he bore his sufferings in solitude and despair, until the abdominal pain and difficulty in uriating led him to seek the aid of Thiandiére, who on making an examination soon discovered the foreign body, but it was so high up that he could scarcely touch it. He endeavoured, but in vain, to extract it with a forceps passed through a speculum. The happy idea then struck him of introducing his hand, which, after having washed out the rectum, he insinuated finger by finger. Conducted by the long branch, he succeeded in reaching the bifurcation of the stick, and disengaged it with difficulty from a fold of the mucous membrane, in which it had become entangled; then, compressing the prongs together, he safely removed it." 64.

In some cases it may be found advisable to divide it with a cutting forceps, or with a small saw, on a wooden gorgeret, after which each half may be removed with ease and safety. Méeck'ren, for instance, mentions a case which occurred to Tholuix, in which the jaw-bone of a fish was situated across the rectum. This surgeon cut it across with a scissors, and then extracted the two portions with ease.

E. *If the foreign body is rough and long,* the surgeon ought, if possible, to fix a ligature on its inferior extremity, and then slip a tube over it; so as to protect the rectum from the violence it would otherwise inevitably suffer from the rough surface during the process of extraction.

*Case.* This is given by Marchetti. Some vicious students of Goettingen introduced into the rectum of an unfortunate woman, all, save the small extremity of a pig's tail, from which they had cut enough of the bristles to render it as rough as possible. Various attempts were made to extract it, but in vain. Marchetti being consulted, adopted a very simple and ingenious procedure, which consisted in securing its inferior extremity with a strong waxed thread, and slipping over it into the rectum a canula prepared for the purpose. He thus defended the bowel from the effects of the bristles, and easily removed it.

F. *If metallic rings, &c., cannot be removed with the finger, blunt hook, or forceps,* they should be divided with a strong cutting plyers, when, in all probability, difficulty will be no longer encountered. Should it be impossible to extract biliary and alvine concretions by means of a lever, the three-branched forceps, or blunt hook, they ought to be crushed with a stone forceps designed for such purpose, and then extracted in fragments.

G. *When Indurated Fæces obstruct the Rectum*, they should be removed with a scoop, or the finger. The sphincter usually soon yields to slow and steady pressure. When the pouch of the rectum is evacuated, a large quantity of warm oil should be thrown up, and the indurated mass thus softened. The patient should be placed as in the position for lithotomy.

H. *Leeches or Ascarides in the Rectum.* The former may accidentally enter the bowel. They will be readily expelled by injecting an infusion of tobacco, or a solution of salt.

Ascarides are occasionally lodged in great numbers between the folds and in the lacunæ of the mucous membrane of the rectum. They give rise to aching, and even lancinating pains in the anus, setting in generally towards the latter end of the day, or beginning of the night. In some instances these attacks are periodical.

Dr. Bushe confines himself to their mechanical removal.

"Brera has recommended this to be accomplished by the introduction of a piece of lard or tallow candle, which, he says, when withdrawn, will bring along with it the greater part of those impacted in the lower part of the bowel. Insertion of the finger, as recommended by Howship, is, however, much more effectual, as we are able to withdraw it in such manner as to extract worms that would elude the lard or candle. The better plan is to use a small lithotomy scoop for the intestine, and that of a director for the lacunæ. This latter instrument I found particularly useful in the case of a boy nine years old, who from his weaning had been tormented with ascarides, and for some weeks had suffered from excruciating pain in the anus, which was greatly aggravated for a few hours after going to bed, attended with fever and slight delirium." 67.

To many of our readers we think that some of the preceding facts will be new, and the surgical directions, perhaps, useful. Both evince much industry, and sound judgment on the part of Dr. Bushe.

### III. Laceration of the Rectum.

Here again Dr. Bushe has the field to himself. Mr. Syme has nothing to say on this subject.

Laceration of the rectum may be incomplete, or it may be complete.

1. *Incomplete Laceration.* This seldom extends beyond the mucous tunic, and, in the majority of cases, is produced by the expulsion of indurated fæces; it may, however, result from the introduction* or extraction of foreign bodies. If the consequence of defæcation, the rent is either transverse or vertical. When transverse, it is situated above the internal sphincter, and is the effect of the forcible extrusion of a fold of the mucous membrane, which, lapping under the mass of indurated fæces, becomes forcibly everted by their expulsion, and in undergoing this change of position, is torn from side to side. When vertical, it generally terminates where the skin and mucous membrane unite, and is the result of forcible and violent distention of the anus.†

* The pipe of the syringe, when awkwardly introduced, may lacerate the mucous membrane.

† In some cases of constipation, while the expulsive muscles act with great

The production of this accident is attended with a painful sensation of tearing and a discharge of blood. The pain, however, gradually, but never entirely, subsides, and is always renewed, with more or less severity, by each successive evacuation. Soon after the accident occurs, inflammation commences, effusion of lymph takes place, the edges and base of the rent become swollen, granulations sprout up, followed by suppuration, and now either cicatrization ensues, or the wound is converted into an ulcer.

The treatment of these cases may be easily summed up. Avoid active purgatives—keep the bowels open by the mildest means, emollient injections, castor-oil, and so forth—cleanse the wound very carefully after each evacuation, a very small quantity of feculent matter lodging in it, being often sufficient to produce extreme suffering—if the wound is very irritable, touch it twice or thrice with caustic, and apply a poultice made with bread and a solution of the superacetate of lead with laudanum—enjoin the horizontal posture—regulate the diet.

Dr. Bushe adds three cases, of which we shall give one. It illustrates the symptoms and the treatment of the affection.

*Case.* " Mrs. C. from whom I removed piles last Summer, sustained a laceration of the mucous membrane, and fine skin of the verge of the anus, in the act of expelling indurated fæces. After suffering great annoyance for five days, she sent for me and complained of pain in the anus, with spasm of the sphincter ani, great nervous excitement and constipation. On examining the parts, I discovered a crevice on the left side of the anus, with an extensive base and tumid edges, between which were two small pieces of indurated fecal matter. I ordered an emollient lavement, tepid ablution, and a saturnine poultice with laudanum, by which her symptoms were mitigated for some hours, but the spasm of the spincter returning with violence during the night, her sleep was broken ; therefore on the following morning, when I visited her, I passed a cane of caustic thrice over the lacerated surface, having previously exhibited an enema and cleansed the parts thoroughly. I then applied a fresh saturnine poultice with laudanum, and enjoined a continuance of the horizontal position. The burning pain soon abated, the spasm did not return, and with the aid of enemata, ablution, saturnine poultices, impregnated laudanum, and the horizontal position, she rapidly recovered." 73.

### 2. *Complete Laceration of the Rectum.*

This is described by Dr. Bushe under three heads, and his account of it is both full and satisfactory.

A. *First Species.* The accident, most commonly designated rupture of the rectum, is in reality nothing more than rupture of the sphincter ani, and is produced by parturition. The circumstances on which it depends, belong either to the mother or the child. Those which appertain to the mother, are, firstly, her position ; secondly, the form of her pelvis, and thirdly, the figure of her perineum.

---

energy, the sphincter remains contracted, and yields but slowly, so that the indurated fæces contuse and abraid the surface of one or more points of the mucous membrane, which, if they do not heal, become converted into fissures.

*Her Position.* When she is allowed to remain in the sitting posture, or to lie on her side, with the lumbar vertebræ curved forward, as in the case when pressure is made against the loins, the head of the child is directed downward and backward, pressing on the rectum and perineum.

*Form of her Pelvis.* When the lower extremity of the sacrum is but little curved forward, as is sometimes the case, the pubeo-cocygean diameter of the pelvis is increased, and in this case, as well as when the sacro-vertebral articulation is prominent anteriorly, the axis of the pelvis passes more backward, consequently, the inclination of the plane which ought to direct the head of the child forward, is diminished, and the arch of the pubis presses it, or, to speak more correctly, gives it a direction downward and backward.

*Figure of her Perineum.* The perineum is occasionally prolonged considerably forwards, so that the vulvular orifice is exceedingly small, and situated close under the arch of the pubes. The majority of cases of this kind are of original conformation; but there are a few purely accidental. About a month ago, in examining an unmarried lady, who was the subject of an immense uterine tumor, Dr. Bushe could scarcely insert his finger into the vagina, and this did not depend upon an obstruction caused by the hymen, but was produced by the prolongation of the perineum. When this state of parts is accidental, it is the result of cicatrization consequent upon laceration or ulceration of the inferior portion of the vulva and perineum. M. M. Buet and Dupuytren each relate a case of this kind resulting from laceration. In Buet's case, sutures were not used, but were in that of Dupuytren. Should a female so formed become impregnated, it will, in all probability, be impossible to prevent laceration of the perineum, which will take place in the median line if this be the weaker part, but if the barrier be equally strong, there may be perforation.

*Case.* "In 1833, I attended a lady, about thirty years of age, in her first accouchement, in whom the perineum was prolonged much forward. Her labour went on kindly until the head of the child came in contact with the external parts, then, the most violent pains, frequently repeated, were scarcely sufficient to propel the child. It was with great difficulty that I was able to prevent the perineum from being extensively lacerated; it however suffered much. Being called to this case in the night, and not expecting any necessity for my pocket case, I did not bring it, else I should have had recourse to a procedure in this instance which, afterwards, I advantageously pursued, under the following circumstances." 76.

Those circumstances were the following.

*Case.* In May, 1834, Dr. Bushe was requested to see a lady. He did not go immediately, and, in two hours, a second urgent message was sent.

"I therefore repaired to her dwelling with all possible speed, and found a healthy young lady, (about twenty,) in labour with her first child, and from the piercing character of her cries, I lost no time in making an examination; when, to my great surprise, I found the perineum much elongated, the orifice of the vagina extremely narrow and close to the pubes, and the head of the child pressing forcibly downward. When the pain, which was violent, subsided, and the head retreated, I introduced my finger, and to my discomfiture, found a rent at least four inches in length, extending transversely through the posterior part of the vagina, about an inch and a half from the vulva. I now inserted my finger into this laceration, and distinctly felt the lower extremity of the rectum firmly contracted. Another pain soon came on, which fortunately was not very strong, so that I was able to support the child's head, while I had a probe-pointed bistoury taken from my pocket case, and when the pain ceased, I divided the perineum obliquely downward and outward on both sides, to an extent sufficient to allow of delivery without further laceration. In this case, I have no doubt had I been a little later, there would have been perforation of the perineum." 67.

*Circumstances relative to the Child.*—These are numerous, and Dr. Bushe mentions only the most common :—The large size and solidity of the head; the rapidity with which it is propelled; neglect in supporting the perineum; omitting to see that the child pursues properly the axis of the pelvis, in proportion as it passes through the external parts ; disengaging the child clumsily and violently with the arm turned towards the rectum, when either the head or feet present ; attempting forcibly to pull the arm corresponding to the rectum, directly downward and backward, instead of bringing it laterally across the concavity of the sacrum, before withdrawing it; neglecting to loop the forceps when placed on the child's head ; leaving them locked and continuing their use after the head has been brought to the external orifice ; making the traction too rapidly and carrying the instruments too far backward, when they are used to bring the head through the external parts ; forgetting when the occiput corresponds to the symphysis pubis to commence elevating the forceps as soon as the head presses on the perineum, so that when this part has passed through the vulva, the forceps will describe a right angle with the abdomen of the mother.

*Extent of laceration.*—As Dr. Bushe has already observed, it commences in the perineum, and never occupies more than the sphincter ani and mucous membrane of the lower part of the rectum. Nor is it likely that it should do more. If an opinion to the contrary has prevailed, that has arisen, in Dr. Bushe's opinion, from the depth of the cleft, resulting from the facility with which these over-distended and naturally lax parts admit of swelling. In consequence of the depth of this cleft, it entangles the fæces, which in such cases are rendered fluid by either medicine or enemata : so that on a superficial examination, a surgeon may easily be deceived as to the real nature of the accident.

b. *Second Species.*—In this, the rupture is above the sphincter, and is sometimes produced by the elbow, or lower extremity of the child, by the crotchet, by forcibly straining to evacuate the rectum, when impacted with indurated feces, but more commonly by the introduction of foreign bodies, particularly of bougies and injecting pipes, these instruments being forced in some instances into the vagina, and in others into the peritoneal cavity. Dr. Bushe once witnessed a case in which the end of an umbrella was projected, in the act of sitting on it, into the rectum, and, passing on obliquely, perforated the recto-vaginal partition.

c. *Third Species.*—"The vagino-rectal partition, sphincter and perineum, are sometimes, though rarely, lacerated. This is most commonly produced by the head or buttocks of the child being directed so far backward, that when the uterus contracts, the recto-vaginal partition is forced downward before the head of the child, and protruded through the dilated anus, and then ruptured from within outward. In 1833, I accouched Mrs. W., the mother of several children, who was in an advanced stage of consumption. Her pains advanced so rapidly, that on my arrival I made an examination, when I found that the head of the child was forcing down the vagino-rectal partition, and had dilated the anus to a great extent. With as little delay as possible, I turned her on her back and gave the head of the child an inclination forward, so that the delivery was safe and rapid." 80.

D. *Treatment.* In the first instance this should be calculated to moderate inflammation. Horizontal posture—enemata—cleanliness, and so forth. When granulations sprout up, the bowels should be as little moved as possible. Dr. Bushe recommends giving laudanum to promote constipation; but it must be recollected that a stool *must* sooner or later be had, and one costive one will do more mischief than many gentle fluid ones. We therefore disagree with him on this point. We would advise liquid nourishment which will not *make* stools—and occasional gentle enemata to bring away what there are, in the best condition for the parts.

"It is only at this period, that sutures should be inserted; for in the many cases I have witnessed, I have never seen one in which union by the first intention took place. This I think may be accounted for by the profuseness and irritating character of the vaginal discharge. When the sutures are inserted in the first instance, they are put upon the stretch by the subsequent tumefaction, and having partly cut their way out, they become quite loose, and consequently are useless during the process of granulation." 81.

When the rupture only extends through the sphincter and mucous membrane, Dr. Bushe does not think the sutures commonly employed sufficient; because, in fact, they do not extend to the bottom of the cleft. Sometimes they answer, but often they do not.

"To obviate the inconvenience which I have ascribed to the interrupted suture, I have devised a pin which is represented in plate viii. fig. iv. This instrument is as thick as that used for hare-lip, and consists of three parts. The first, which is made of silver, is from one and a half to two inches long, curved as represented in the plate, terminating at one end in a female screw, and at the other in a transverse shoulder about a quarter of an inch long. The second is a triangular steel pin, exactly resembling that used for hare-lip, and screws into the extremity of the first portion. The third is made of silver, and resembles the transverse shoulder of the first portion, with this exception, that a small male screw passes vertically from its centre, so that it may be fixed into the first portion, when the second is removed. This instrument is to be used in the following manner: the first and second portions being united, provided the tumefaction has nearly subsided, and granulations are formed, the patient should be brought to the edge of the bed, her hips elevated, and her knees approximated and carried towards the chin. The parts being now cleansed, the needle ought to be dipped in oil and inserted into the left side of the perineum, a line more than half the breadth of its curve from the edge of the wound, and immediately above the verge of the anus. When it has passed vertically for a distance equal to two-thirds of the depth of its curve, its point should be projected transversely, so as to cross the bottom of the wound, and then carried outward through the other side of the perineum. This stage of the operation will be greatly facilitated; firstly, by pressing out the left labium during the transmission of the needle through the left portion of the perineum and the base of the wound; and secondly, by steadying the right side of the perineum, with the extremity of the thumb placed immediately without the point through which we desire the needle may pass. When the puncture has been completed, the steel pin shoul be unscrewed and the third portion fixed in its stead. If it be thought advisab to insert a smaller pin higher up, it may be done, and then a thread should b twisted over their extremities, as in hare-lip. It may be prudent to place a lig bolster of lint beneath the twisted ligature. This method of operating was fir carried into effect in the alms-house of this city, by my friend Dr. Stevenso who, not only on this, but on other occasions, afforded me opportunities of tes ing chirurgical innovations." 83.

The principle of this "innovation," and the nature of the pin, are s obvious from mere description, that we do not think it necessary to subjo a wood-cut. The idea appears to be a good one. Dr. Bushe relates a cas in which he operated with complete success on a lacerated perineum of fiv months' standing.

When the rectum is ruptured above the sphincter, provided the rent b not large, it will generally heal: firstly, by keeping the patient on a meag diet, enforcing the horizontal position, and relieving the bowels with ene mata, provided the peritoneum be not lacerated, and with castor oil if it is and secondly, when suppuration is established, by exhibiting opium to qui the bowels for a few days. If, however, the wound does not heal, its edg should be pencilled with lunar-caustic. In the case above alluded to, i which the rectum was torn by the extremity of an umbrella, the edges of th wound required three applications of caustic before they adhered. Dr. B admits that such cases may require the actual cautery.

When the recto-vaginal partition, sphincter, and perineum are tor through, the case is serious. In one bad case he operated with success b means of the perineal pins already described, small curved needles, and th *pince porte aiguille* of Dieffenbach.

Dr. Bushe is not inclined to attach much value to the plan lately adv cated by some surgeons in this country, of dividing the sphincter ani i cases of laceration of the rectum.

### IV. Inflammation of the Rectum.

Mr. Syme has nothing to say upon this. Dr. Bushe has not a great deal There is not, in reality, much to be said.

It is usually produced by causes so obvious that we shall not enumerat them. But we may observe that, in two cases, Dr. Bushe, *thought* he trac it to gout; and he has found it very common in coachmen, from sitting o cold and wet seats in Winter. He does not mention erysipelas as a caus yet it is not an uncommon one. The symptoms, as we know by observatio ourselves, are both severe and formidable. Dr. Bushe's description coincid with what we have personally witnessed.

The disease, he writes, is manifested by a sense of fulness, weight, burn ing and throbbing in the fundament, which is increased by sitting ere The act of defecation is accompanied with and followed by severe pai which, from the contraction of the sphincter ani, assumes a spasmodic cha racter. The heat of the intestine is much increased, as may be ascertain by the introduction of the finger, which, however, is attended with horrib suffering. There is considerable fever. The urinary organs sympathize there may be dysury, strangury, or even retention of urine ; the first and second of these arising from actual inflammation of the *trigone vesicale*, and the third from spasm of the perineal muscles. After the disease has continued for some time, the cellular tissue external to the rectum becomes engorged, and, if the primary affection be not removed, suppurates. Occasionally, the peritoneum becomes inflamed, and the patient's suffering and danger are thereby much increased ; but, fortunately this is a rare occurrence. In some cases, particularly in robust persons beyond the meridian of life hæmorrhage occurs from time to time, especially after stool, and invariably brings relief. The inflammation sometimes extends to the colon, and then a new series of symptoms sets in, viz : tormina, tenesmus, muco-sanguinolent evacuations, and a considerable increase of fever. Females are sometimes tormented with bearing-down pains, and profuse mucous discharge from the vagina. It not unfrequently happens that, after the disease has continued a few days, an abundant purulent secretion takes place, with which the pain, burning and throbbing subside, the febrile symptoms disappear, and complete restoration rapidly ensues. Some practitioners are alarmed at the appearance of this discharge, supposing that it indicates an extension of the inflammation to the colon ; therefore, it is well to know that the fæces are of their natural aspect in inflammation of the rectum, while in dysentery they are mixed up with blood and mucus.

The worst cases, that have fallen under our own observation, have arisen from the extension of erysipelatous inflammation to the bowel. Such cases are too often fatal. The patient has extreme anxiety—frequent but not strong pulse—loaded, and often dry tongue—hot skin—perhaps delirium. The belly is tympanitic, and there is obscure pain upon pressure, especially in the hypogastric and the pubic regions. Sometimes there is vomiting, in all cases there is purging, with great pain in the bowel for the most part, when the stool passes. These are the chief symptoms. If the patient recovers they decline—if, as often happens, he dies, typhoid symptoms set in, and on examination, after death, there is either diffused redness of the intestinal mucous membrane, or diffuse inflammation of the pelvic cellular membrane, or, which is uncommon, there is peritonitis.

The treatment consists in removing, if possible, the cause of the disease. The remedies will in some degree be determined by that necessity. General blood-letting *may* be requisite—leeches round the anus are more often proper. Diluents—sitting over the vapour of hot water—opiate enemata and poultices—fomentations to the abdomen—in some instances calomel and opium, are remedies which will probably suggest themselves to the well-informed practitioner. When the affection results from erysipelas, depletion must be avoided.

When women suffer from bearing-down pains, anodyne enemata and warm hip-baths afford them most relief.

Strangury and dysury require no other treatment than the warm hip-bathing ; but should there be retention of urine, we ought, in addition to the remedies already mentioned, exhibit a full dose of morphine. In case this fails, a solution of tartar-emetic should be given every ten minutes until it produces vomiting ; when, if it does not produce the desired effect, as a last resort, a gum-elastic catheter ought to be cautiously introduced.

### V. INFLAMMATION AND EXCORIATION OF THE ANUS.

They occur more often in the corpulent than in the lean—in gross livers, than in temperate ones. Walking, riding, hot-weather, irritating secretions, bring them on.

The general treatment should improve the secretions.

The local consists in cleanliness—wash of zinc or Goulard—absorbent powders—or sedative ointments. The best we know of is the following:—

℞ Unguenti Zinci,
Cerati Plumbi, āā ℥ss.
Acidi Hydrocyan. Med. ♏ v.
M. ft. Unguent.

The parts should be first bathed with tepid goulard water, and then this ointment should be applied to them. With the following suggestions of Dr. Bushe, we pass to another subject :—

"When excoriations arise from want of cleanliness, the hair and discharge become matted together, and thus form a crust, which covers the excoriated surface. Under such circumstances, the parts ought to be poulticed until the crust becomes so soft that it can be removed without cutting the hair, for should this be done, as I have once seen, the irritation created by the stumps will increase the inflammation, protract the healing of the suppurating surface, and render the patient exceedingly uncomfortable, until the hair has again acquired sufficient length to diminish the friction of the buttocks on each other. After the parts are sufficiently cleansed, a saturnine cataplasm impregnated with laudanum should be applied, and changed every six hours, at which time the diseased surface ought to be washed with cold water and the common yellow soap." 94.

### VI. INFLAMMATION OF THE RECTUM AND ANUS ARISING FROM THE APPLICATION OF GONORRHŒAL MATTER.

Our author's sentiments on this head may be comprised in one short quotation.

"In cases of this kind, in addition to the symptoms of inflammation mentioned in the two last chapters, there is profuse purulent discharge from the commencement, which produces excoriation of the anus, and, in some instances, of a considerable portion of the adjacent parts.

In the treatment of this disease, besides the means recommended in the chapters alluded to, injections ought to be employed. Those of the superacetate of lead, sulphate of copper, and more especially of the nitrate of silver, in the proportion of five or ten grains to an ounce of distilled water, are the most appropriate."* 97.

This must be admitted to be a very imperfect account of a not unfrequent, and, at times, a very troublesome affection. We will add what will tend to make the description more complete, although we shall only touch on the more important circumstances.

---

"* Velpeau recommends Howard's calomel in a decoction of marsh-mallows, in the proportion of a drachm to an ounce. He also entertains a good opinion of a white precipitate ointment. (Dict de Méd.)"

Gonorrhœal inflammation of the extremity of the rectum is more common, for obvious reasons, in females, than in males. It is usually of a chronic character, at least we have never seen it acute. The patient seldom complains to the surgeon until there is excoriation. If observed before that stage, there is itching and uneasiness felt at the anus, and pain on going to stool, especially if this is costive. If the surgeon examines the rectum, it appears red in converging streaks, or rather in streaks stretching into the gut. On minutely examining the inflamed surface, it will be found in parts deprived of its epithelium, and secreting a kind of mucus.

At a later period, the excoriations have become ulcers. These are usually superficial, yellow on the surface, with a thin red edge sometimes undermined, and a moderate discharge. At the time of stool, especially if this is at all a confined one, the pain felt in the ulcer is excessive.

At other times the excoriations, instead of ulcerating and forming sores more or less excavated, are attended with interstitial deposition into the subjacent cutis, and form superficially ulcerated condylomata. These are found in the perineum, and especially at the verge of the anus. The colour is of a dirty red, with an irregularly whitened surface; the latter appearance being due to the adhesion of the discharge of the superficial ulceration. The condylomata are in general not painful, unless they encroach so much upon the anus as to be stretched or torn by the passage of the fæces. By the patients they are not unfrequently mistaken for piles, and an ill-informed surgeon may commit the same blunder.

The treatment of the excoriations, before they become actual ulcers, is simple. Cleanliness—black-wash—the diminution and removal of the exciting cause, the gonorrhœal discharge, and the prevention of its contact with the parts affected—and smart purging are usually sufficient.

The ulcers require, in addition to such means as are calculated to remove the gonorrhœal complaint, the application of lunar caustic to themselves. This should be employed every second or third day, and the bowels should be kept gently opened by means of castor oil. A dose or two of calomel and opium, and, if necessary, an opiate suppository or enema, tend greatly to assist the lunar caustic in removing the excessive irritability of the sore.

The excoriated or ulcerated condylomata are often troublesome. The best local application is, in most instances, the solution of oxymuriate of mercury. The strength may vary from half a grain to two grains to the ounce. If ulcerated, they may be touched, at intervals, with lunar caustic. It is often requisite to put the patient under the influence of mercury, in order to remove the condylomatous thickenings.

Such are the brief observations we would offer on these matters.

### VII. Fissure of the Anus.

We cannot yet introduce Mr. Syme to our readers, but must still confine ourselves to Dr. Bushe. The affection, we are now about to consider, is commonly known in this country under the name of ulcer of the rectum. It is pretty common, very painful, difficult to treat, and liable to be overlooked by a surgeon ill-acquainted with its symptoms and its seat, comparatively easily managed, and still more easily detected by a surgeon well acquainted with its characters. On these accounts, we shall devote some little attention to it. Dr. Bushe's description is very circumstantial, and very accurate.

"The disease," he says, "is an ulcer, about the eighth of an inch in breadth, and from a quarter to an inch in length, situated immediately within the anus, generally on one or both sides, occasionally on the posterior, and still less frequently on the anterior part of the aperture. In the majority of cases it is confined to the mucous membrane, though occasionally it extends to the muscular tissue. Its inferior extremity generally corresponds to the edge of the external sphincter, though sometimes it is placed a little higher up or lower down. The base of this oblong ulcer is generally red, but sometimes grey, in consequence of the deposition of lymph. When recent, its edges are soft, pliant, and but little elevated; in proportion, however, as it becomes chronic, so are they more hard and prominent, changes which depend upon the interstitial deposition of adventitious matter from the irritated capillaries.

The surrounding mucous membrane is in its natural state in some cases, particularly when recent, but not unfrequently it wears an erysipelatous hue, and again assumes a livid aspect, and becomes soft.

Women are more subject to this affection than men, which arises from their leading more sedentary lives, and, consequently, being more subject to constipation of the bowels. It generally occurs in the meridian of life; nevertheless, I have treated a case in a girl of eighteen, and another in a woman of sixty-nine years of age.

In the majority of cases it is preceded by vascular tumours of the rectum; then it is situated between two of them, and is produced by the forcible passage of indurated feces. In this act the vascular tumours are first prolapsed, and then separated, during which process the mucous membrane, rendered friable by inflammation, is ruptured. The contraction resulting from operations performed in this region, and the spasm of the sphincter, by opposing the free egress of the feces, become a frequent source of fissure in the former, by disposing to rupture, and in the latter by contusion and abrasion of the mucous membrane.

In the three different instances I have mentioned, the laceration of the mucous membrane does not heal, because the primary affection still continues, and even in some instances, as heretofore explained, (see chapter v.) the rupture is converted into an ulcer, though no primary affection existed.

Besides the causes now specified, inflammation, and consequent abrasion may, from the columnar arrangement of the mucous membrane of the lower extremity of the bowel, give rise to one or more fissures." 101.

The symptoms are the following:—In the commencement of the disease, they are not severe, being merely, at one time, a pricking or stinging sensation, at another, a slight smarting in a certain point of the anus, which, under the use of lavements and low diet, subsides either altogether, or, after a few days, returns with some severity. The pain, gradually increasing, becomes burning, sometimes lancinating, and when severe, throbbing. It is increased by forced expirations, as coughing, sneezing, and urinating. Every effort to discharge gas and fæces, is attended with excruciating torment, which continues for one or more hours, attended with violent spasmodic action of the sphincter ani. So violent is the agony, that most persons thus afflicted put off the calls of Nature, maintain the recumbent position, and some even avoid taking a proper quantity of nourishment, for fear of increasing the fæcal mass. The pain is always increased by stimulating food, and in females during menstruation. Occasionally Dr. B. has seen it assume a periodical character, which depended upon some peculiar state of the constitution. When the fæces are solid, they are slightly streaked with blood and matter, and when more soft, are figured and of small size.

Of course the actual existence and precise seat of the fissure are only to be ascertained by inspection and examination. For this purpose the buttocks should be forcibly separated, when the inferior extremity of the fissure will be brought into view; but in some rare instances we cannot accomplish this object, in consequence of its elevated site, and we are compelled to trust either to the introduction of the finger, or to the dilatation of the anus with the speculum for its detection. In a few cases, though it is immediately discovered upon separating the buttocks, we can only ascertain its length, by the means just mentioned.

The introduction of the finger is attended with great difficulty and torture, particularly when pressure is made on the fissure, which seems, in some instances, to be a mere depression, in others, to be surmounted by pretty high edges, while in a few rare instances, we only become cognizant of its situation by the increase of suffering in a certain point, under the same amount of pressure.

When the pain is violent during and after stool, it is accompanied with fever; and when it continues for any length of time, emaciation, hypochondriasis, and an irritable state, with a severe train of nervous symptoms, ensue.

*Treatment.*—On this head, Dr. Bushe's observations appear to us so satisfactory, that we transcribe them in full.

"The patient should be kept on a low diet, and confined to the recumbent position. The common practice of administering cathartics, so as to produce fluid evacuations, cannot be too highly censured,—for such discharges stimulate the ulcerated surface, and thus induce dreadful irritation and spasmodic contraction of the sphincter ani; therefore, the better plan is to administer daily an enema of flax-seed tea, and after its operation to cleanse the parts well. If the disease be mild, the application of the unguentum acetatis plumbi will be sufficient for its healing, and if there be much spasm of the sphincter, the extract of belladonna will prove a powerful auxiliary. Dupuytren recommended an ointment of this kind, the proportions being a drachm of the lead, and the same quantity of the belladonna, to six drachms of lard. Before I became acquainted with his practice, I was in the habit of applying the nitrate of silver to superficial fissures attended with spasm, and then introducing meshes of lint, besmeared with a mass consisting of one part of the extract of belladonna, and seven of spermaceti ointment, a course of practice which has succeeded when Dupuytren's ointment has failed.

When a fissure will not heal under this treatment, and the patient continues to suffer, we should no longer delay the division of the sphincter, which never fails to give immediate relief, and to effect a rapid cure.

To perform this operation, the patient should be placed opposite a window, couched on his side; an assistant ought to separate the buttocks, and retain them so during the operation. The surgeon having oiled the fore-finger of his left hand, inserts it into the anus as far as the second joint, and uses it as a conductor for the knife, delineated in plate viii., fig. iii., whose blade is two inches long, and one eighth broad, with a blunt extremity. Having passed the blade flatwise as high as the superior border of the internal sphincter, he then turns its edge towards the fissure, provided it be on the side of the bowel, and divides both sphincters, by cutting from within outwards, gradually increasing the pressure so as to ensure the complete section of the external muscle. Provided a fissure exists on the opposite side, it ought to be treated in the same manner. When the posterior or anterior portions of the intestine are the seat of disease, as the division of the sphincter and not the fissure is the desirable object, the incision ought to be made on the side; because in this way, the external sphincter can be safely, yet perfectly, cut across, and the inconvenience arising from the shortness of the space between the coccyx and verge of the anus, the proximity of the bulb of the urethra in the male, and the shortness of the perineum in the female, is avoided. But there is also another objection to the performance of this operation in the median line, viz. the difficulty in healing wounds in this situation, in consequence of the friction created by the motion of the inferior extremities. After the hemorrhage ceases, dossils of lint should be placed in each wound, and secured by a compress and T bandage. A full dose of morphine ought to be exhibited, and nothing but toast-water, broths, gruel, and the like, allowed for two or three days. The dossils of lint, compress and bandage, should then be removed with great care, the bowels washed out with an emollient lavement, and fresh dressings applied. This course ought to be pursued daily, gradually diminishing the size of the dossils of lint, until the wounds heal, which will be in about three weeks." 107.

Dr. Bushe relates four cases; but, though they illustrate the effects of treatment, they are not necessary for its comprehension. We therefore omit them.

### VIII. Neuralgia of the Extremity of the Rectum.

Dr. Bushe suspects that, in the majority of cases described by authors, in which both the anus and genito-urinary organs were said to be the seat of neuralgia, no such disease affected the anus, but that, in consequence of irritation in the genito-urinary apparatus, the sphincter ani was thrown into a state of painful contraction. Dr. B. relates the "only genuine" case of neuralgia commencing in the genito-urinary organs, and from thence extending to the extremity of the rectum, which has come within his observation.

*Case.* A physician of middle age, active habits, and tolerably good health, but of a nervous temperament, was subject to occasional attacks of neuralgia of the face, stomach, and testicles. Twice or thrice in the year, he would be seized with pain over the pubes and a desire to micturate, which generally subsided in twelve or twenty-four hours. More rarely, he suffered excruciating pain in the end of the penis, or in the posterior part of the urethra, attended with a similar state of the extremity of the rectum, but without contraction of the sphincter. These attacks were generally either preceded or followed by neuralgia elsewhere. He tried various remedies without advantage, and at last contented himself with pressure during the paroxysm, which he thought was always considerably moderated by this expedient.

Our author details three cases in which the neuralgia seemed to be confined to the extremity of the rectum. These cases are interesting, if not practically important; and we shall notice them.

*Case* 1. In 1829, Dr. Bushe was called to see Mrs. H., a nervous lady, about thirty years of age, who, for several months, had suffered from lancinating pain in the extremity of the rectum. For weeks this pain would be very severe, and then nearly, but not altogether, subside. Her distress was greatest towards the close of the day, and then she was compelled to go to bed and take *black drop*. Changes of temperature had a baneful influence on her, not only increasing the pain in the anus, but rendering her restless and melancholy. Her bowels were generally constipated, to remedy which she generally took three doses of magnesia every week. During defecation her distress was very much increased, especially when the excrement was hard. Dr. B. examined the parts with great care, but could not detect any organic lesion. There was no spasm of the sphincter, and she bore pressure on every part of the rectum that the finger could reach without pain, save on a spot about the size of a shilling on the left side of the intestine, rather less than half an inch above the verge of this orifice, which was so exquisitely tender that she screamed out when the finger was pressed against it. He recommended her to try anodyne suppositories, blue-pill, and the carbonate of iron; but never learnt the result.

*Case* 2. In 1831, Dr. Bushe was consulted on the case of Mrs. E., a delicate lady, of nervous temperament, aged twenty-five. She informed him that for three years she had laboured under some disease of the anus, which commenced insidiously, and without any known cause. She had become irritable, desponding, and emaciated. Her bowels were never moved without medicine, which she had recourse to every alternate day. During the fæcal discharges, and for some time afterwards, she invariably suffered the most excruciating pain, which was attended with involuntary contractions of the sphincter. For some months past, she was never entirely free from pain, and for weeks together it assumed a periodical character, gradually increasing every afternoon, and then mitigating in about three or four hours. Atmospheric changes never failed to affect her,—the pain, when stormy weather was about to take place, being most agonizing. While suffering severely, Dr. Bushe made an examination of the parts, but could not discover any structural derangement; however, the sphincter was so forcibly contracted, as to render the introduction of his finger not only exceedingly difficult, but painful; and one point of the gut, a few lines above the anus, on the right side, was so tender that, when he touched it, she was thrown into the greatest agony.

"I therefore carefully examined the parts, thinking that I might detect a fissure, but my conjecture was not realized, for the painful point in every respect appeared perfectly healthy. I endeavoured to relieve her by purgatives, iron, quinine, arsenic, lavements, opiate suppositories, and the introduction of meshes of lint besmeared with seven parts of spermaceti ointment, and one of the extract of belladonna; but without effect. It then occurred to me that an incision carried through the painful part and the sphincter, was a feasible means of relief. I communicated my views of the case to Mr. E., who consented to the proposed operation, which I performed with perfect success." 115.

*Case* 3. In 1833, Dr. Bushe was consulted on the case of Mrs. W.

"She was about nineteen years of age, of a fragile constitution, exceedingly nervous, and for seven years had occasionally been the subject of neuralgic pains in the face. She had been married between five and six months, and pregnant for three; during the last six weeks, she was seized every two or three days with violent lancinating pains in the anus, attended, when severe, with alternate contraction and expansion of the sphincters of the most forcible and sudden character; the latter being productive of a discharge of mucus mixed with a small quantity of blood, and sometimes with feces. During these attacks she was in the habit of folding a napkin into as small a compass as possible, placing it between her buttocks, and sitting on a wooden chair.* By this expedient she found that her suffering was greatly mitigated, though she was constantly compelled to have recourse to morphine. I tried various remedies, as iron, quinine, arsenic, anodyne enemata, &c., without effect; however, upon quickening, the pain ceased, and did not return until last Spring, when she was again pregnant; but upon her miscarriage, which occurred on the third month, it again vanished.

I may mention, that after this lady weaned her child, I have seen her nearly deranged with neuralgia of the face.

This case is not exactly like either of the preceding, as there was no part of the extremity of the bowel more affected than another, and the pain, which was not constant, instead of being increased, was relieved by pressure; yet that they were all cases of neuralgia, there can be no doubt."† 116.

### IX. Spasmodic Contraction of the Sphincter Ani.

This subject is discussed in the eleventh chapter of Dr. Bushe's work, and in the sixth of Mr. Syme's. In the notice of the symptoms and effects of fissure of the anus, allusion was made to spasmodic contraction of the sphincter as one of them. In the present instance the latter affection is to be regarded in the light of a more substantive affection.

### A. *Simple Spasmodic Contraction of the Sphincter Ani.*

*Symptoms*—Mr. Syme's account of these is not a bad one.

---

* "Montegre, when treating of the character of these pains, says, 'Et alors chose remarquable, la compression les soulage.' Velpeau has expressed the very opposite opinion, (Dict. de Med. ed 2d. tome iii. p. 282.) As these cases demonstrate, both authors are in error, and the cause has been the same in both instances, viz. an avidity to draw general conclusions from isolated facts."

† "Mr. Mayo relates the following curious case of this disease: 'I attended a patient with Mr. Stephenson, of the Edgeware-road, who suffered from pain in the rectum. Something less than two years before this, he had a syphilitic ulcer on the penis, for which he had taken an unusual large quantity of mercury, owing to the difficulty of producing sensible mercurial action on his system. The ulcer, however, healed; but while he was recovering, and his system was yet charged with mercury, he began to experience aching pains in the incisor teeth and in the rectum. The sense of aching in the teeth and in the rectum was not constant, but would come on frequently during the day, without any assignable cause. It had lasted a year and a half, during which he remained perfectly free from symptoms of lues. The patient, who was otherwise in good health, suffered his mind to be greatly distressed by the continuance of the neuralgia. He was anxious to try every plan which held out the least promise of benefiting him. But of all the remedies which he tried, he appeared to experience relief from one only, which was a course of sarsaparilla.' (Observations on Injuries and Diseases of the Rectum. London, 1833, p. 56-7.)"

"Cases," he says, "are occasionally met with in which the patient expresses great suffering from uneasy feelings referred to the neighbourhood of the anus, though no alteration of structure in the parts concerned can be detected by the most careful examination. It is stated that the bowels are evacuated with difficulty and pain,—that the pain frequently does not come on until after going to stool, and then continues extremely severe for an hour or longer;—that sitting is very uncomfortable, unless the body rests on one hip, so as to protect the anus from pressure,—and that there is an unpleasant sensation of fulness in the perineum, with heat in the urethra, frequent desire to make water, or other symptoms of irritable bladder. These complaints are not always equally severe, and often become greatly aggravated, from time to time, with more or less complete remissions, which are not unfrequently preceded by discharges of blood or matter from the rectum. The anus, instead of presenting its ordinary conical appearance, looks flat when examined, and hardly presents any trace of the orifice, owing to the inordinate contraction of the external sphincter muscle. If the finger be introduced, which is not accomplished without great pain and difficulty, every attempt to examine the gut causing excessive distress, not only at the time, but for hours afterwards, it feels much more strongly compressed than usual. And when the nates are held aside, so as to bring the lining membrane of the anus into view, one or more ulcerated fissures are occasionally observed between its folds.

This affection occurs in every rank of life, but is almost entirely confined to the male sex. From not being attended with any obvious alteration of structure, it is often considered imaginary, and treated merely as a nervous complaint. It has only of late years attracted the attention of the profession, and is yet far from being familiarly known to practitioners in general. Boyer has given an excellent description of the disease, under the title of Fissure of the Anus, believing that the excessive contraction of the sphincter depended upon the irritation of the ulcerated chops, which he thus designated. That these two morbid affections frequently exist together there can be no doubt. But that the spasmodic stricture is of secondary origin, and dependent upon the other, is not reconcilable with the facts presented in practice. In a considerable proportion of cases, I have found the sphincter firmly contracted, without any perceptible fissure or abrasion of the surface. And I have also, though more rarely, met with fissures producing great uneasiness to the patient, but not accompanied with spasmodic stricture of the anus." 135.

It will be observed that Mr. Syme has in some measure mixed up the two affections—fissure of the anus, and spasmodic contraction of the sphincter. They may co-exist, or they may not; and while their occasional co-existence should be noticed, their individuality should be made, as Dr. Bushe has made it, a subject of special consideration. We have seen many instances of spasmodic contraction of the sphincter unaccompanied with fissures, or with any other appreciable alteration of the gut.

*Causes.* Mr. Syme observes that it is very difficult to explain the cause or origin of this complaint. Its nature leads to the suspicion of some chronic excitement or irritation of the parts concerned, or those in their neighbourhood. Anxiety and distress of mind have evidently a powerful influence in confirming and aggravating its symptoms, and may not improbably also occasion its commencement. General irritability of the system may also constitute a predisposition to its production. And every thing that tends to irritate the rectum is of course apt to increase the patient's sufferings. Thus, introducing the finger or foreign bodies of any kind within the anus—forcibly expelling indurated matters from the bowels,—using stimulating articles of food or drink,—and remaining long in a sitting posture, are observed to be hurtful.

So far as we have seen, the affection has occurred in persons of a nervous and irritable habit, and has, apparently, been traceable either to costiveness of bowels or to irritation of the genito-urinary organs.

*Treatment.* Mr. Syme has only one remedy to propose, or, at all events, he only proposes one—division of the sphincter.

"In the treatment of this spasmodic stricture, it has been found that the most effectual, if indeed not the only means of affording relief, consists in making an incision through the constricted parts. Boyer recommended this operation as essential for the cure of fissures at the verge of the anus, which he considered the cause of the contraction. And though his theory in this respect seems questionable, the advantage of the practice cannot be disputed. But the good effects of an incision are no less remarkable when there is merely contraction without fissure; and therefore, in a practical point of view, it is of little consequence how the two affections are supposed to be connected.

Boyer believed that it was necessary to cut through the whole thickness of the sphincter; and instructions for performing the operation to this extent have been given by many later writers. From my own experience, however, I am satisfied that it is not necessary to divide more than the external sphincter, or merely a portion of it, together with the lining membrane of the anus and subjacent cellular substance. The most convenient instrument for the purpose is a blunt-pointed straight bistoury, which may be guided on the finger with a sawing motion to the requisite depth. The incision should be made towards the side of the gut, and through one of the fissures, if there be any present. A piece of dry lint is the only dressing required after the operation; and the wound may be treated subsequently as if it had been made for a *fistula in ano.*" 137.

No doubt, in extreme cases, division of the sphincter is the only remedy of permanent utility. But certainly there are some instances of spasmodic contraction of the sphincter, for which no such operation is requisite. In three instances, we have cured the complaint by hip-baths—suppositories of belladonna and opium—gentle laxatives, such as castor-oil with tincture of henbane, or with laudanum—the horizontal posture—and a regulated diet.

Dr. Bushe relates a case which did well under mild treatment.

*Case.* Mr. N. a robust young man, and a high liver, consulted Dr. B. with a view of obtaining relief from a forcible, painful, and, sudden contraction of the sphincter ani, which occasionally aroused him from his sleep at night, and generally lasted two or three hours. He had been subject to these attacks for eight or ten weeks, and altogether may have had a dozen seizures, for which he could assign no cause. Dr. B. prescribed a vegetable diet, exercise, a warm hip-bath every other night, three grains of blue-pill with one of ipecacuanha at bed time, and a tea-spoonful of Epsom salts in two ounces of quassia tea, on the following morning. Under this treatment he soon recovered, and has not had a return of the disease.

In another case, we are not told that treatment was of service, but the case itself is interesting.

*Case.*—Dr. ——, an eminent physician in New York, exceedingly muscular, of a nervous temperament, and very subject to mental depression, informed Dr. B. a short time since that, for seven years past he has been seized, while in his cabriolet, about every three months, with violent contraction of the anus, attended with almost insupportable suffering, which however, soon subsided; that he was occasionally attacked during the night with paroxysms of much longer duration, but of a less distressing character; and that his fæces, when solid, were of smaller dimensions than natural.

The following cases were not relieved without division of the Sphincter.

*Case.*—In November, 1829, Dr. Bushe was called to see Mrs. Q., who was labouring under what she had been informed was stricture of the rectum. She informed him that in April she had a diarrhœa, after which her bowels became constipated, and a train of symptoms exactly similar to that mentioned in the chapter on fissure set in, save that the fæces were neither streaked with blood nor matter. She had become emaciated, restless, feverish, and depressed in mind, with a firm conviction that her end was drawing near. On examination, Dr. B. could not discover a fissure, nor that any one point was more tender than another; but the anus was closed so firmly that he could only enter his finger with difficulty. He prescribed a light nutritious diet, hip-baths daily, a table-spoonful of castor oil every morning, and the introduction of meshes of lint, besmeared with one part of the extract of belladonna, and seven of lard. As she did not improve under this course, he substituted the bougie for the lint; but so painful did this remedy prove, that he had to abandon it. Then, he divided the sphincter as in fissure, the result of which was a rapid cure.

The last case of simple spasmodic contraction of the sphincter which we shall extract, is related by Mr. Syme.

*Case.*—"I was asked," says Mr. S., "to see a gentleman about sixty years of age, who stated that, a few weeks before, after sitting out a long debate in the House of Commons, he had felt extreme difficulty in evacuating the bowels, having previously for several years experienced more or less uneasiness from this source; that he had consulted a physician and surgeon in London, who prescribed laxatives without affording relief; and that his complaint had continued so as at length to confine him to bed. I proposed an enema, which was at once objected to, on the ground that the anus would not admit the smallest-sized tube. Suspicion being thus excited, the anus was examined, and found to present the characteristic features of spasmodic stricture. Having explained my views of the case, I gently insinuated the narrow sheath of a *bistoury caché*, which I happened to have with me, and then expanding the blade, withdrew it, so as to make an incision at one side of the orifice. A copious stool immediately followed, and the patient was at once completely relieved from his complaint." 138.

B. *Spasmodic Contraction of the Sphincter Ani, with Affection of the Genito-Urinary Organs.*

On this Mr. Syme makes no observations, but Dr. Bushe relates several interesting cases.

"Mr. O., a grocer, twenty-eight years of age, robust and of good habits, in 1830, being about to enter into the matrimonial state, consulted me for an affection of the urinary organs, which, from his account, I took to be stone in the bladder. I therefore sounded him very cautiously, but without being able to find a calculus. As he complained of spasmodic pain in the lower extremity of the rectum, especially when the urinary symptoms were severe and for some time after stool, I proceeded to examine this intestine, when I found the sphincter firmly contracted; however, all the parts in this region were perfectly healthy. The pain resulting from the examination of both these organs was very distressing, though the greatest possible gentleness was observed. He took pills of henbane valerian and white oxide of zinc, according to the formula of Meglin, and inserted suppositories of opium and belladonna at bed-time, while his bowels were kept easy with lenitive electuary or oil, and I injected his bladder daily with a solution of gum arabic and opium. His improvement was very slow, and as I found his urine to be surcharged with lithic acid, I put him upon vegetable food, ordered soda in sufficient quantity to correct the acidity of his urine, and a warm bath daily. By pursuing this course steadily for a few months, wearing flannel next his skin, and sedulously avoiding cold, he entirely recovered, then married, and since has had no return of the disease." 121.

It is evident that the symptoms, in this instance, depended on an acid condition of the urine, and on that irritability of the bladder induced by it.

*Case* 2.—In 1831, Dr. B. was called to Mr. M., who for five years had been afflicted with symptoms of stone, and was repeatedly sounded, both in this country and in Europe, by distinguished surgeons. When Dr. B. saw him, in addition to the ordinary symptoms of stone, he had close and painful contraction of the sphincter ani; which he informed Dr. B. had only set in within six months, and occasionally was almost insupportable. His urine was loaded with acid, and deposited much mucus. Being a man of fashion, he was frequently exposed at night to cold air, from which he suffered dreadfully; indeed, if from missing his carriage, he was compelled to walk home at night, he never failed to have a marked change for the worse in all his symptoms. For some time he took the pills of Meglin without any advantage. Dr. B. then injected a solution of opium and gum water into the bladder, and ordered a liniment containing the extract of belladonna to be rubbed over the pubes and perineum. This plan of treatment being no more efficacious than the first, he consented to try the course which Dr. B. first recommended, viz: Confinement to the house except when the thermometer was above 60°, vegetable diet, flannel next the skin, warm baths, lenitive electuary or oil when the bowels were out of order, soda to neutralize the acid state of the urine, and an infusion of buchu as long as a mucous sediment was deposited by the urine. By following up this course for about nine months, he entirely recovered.

*Case* 3.—Mr. A. applied to Dr. Bushe, on account of a hydro-sarcocele. Remedies, which we need not particularize, were ordered for this complaint. As they were not productive of the benefit desired, Dr. Bushe examined the urethra. While passing a steel sound in the most gentle manner, though a very reasonable person, he screamed violently, and the spasm of the canal was so great, that he could not pass the instrument into the bladder. Dr. B. ordered him a warm hip-bath, and an ounce of castor oil, with twenty-five drops of laudanum. As no bad symptoms followed this attempt to introduce the sound, he made three more essays, on alternate days, with no better result, the same irritation being manifested on each occasion. In the evening of the last day, on which he endeavoured to pass the instrument, he sent for

Dr. B., complaining of excessive irritation of the bladder. Dr. B. ordered warm bathing, marsh-mallows syrup with water, and a dose of morphine. He soon obtained relief; however, on the following day, he had a similar attack, which yielded to the same means, but returned at midnight. In his distress, he had a carriage called, and drove to Dr. B.'s house, complaining dreadfully. Dr. B. recommended him to repeat the bathing, to continue the marsh-mallows syrup with water, and to take, according to circumstances, one or two enemata, composed of four ounces of the mucilage of starch, and forty drops of laudanum. At nine o'clock in the morning he wrote Dr. B. a note, beseeching his attendance, as his suffering was dreadful. When Dr. B. arrived, he found him moaning, and the tears flowing down his cheeks. He said that a short time after taking the injection, he was seized with violent pain and contraction of the anus, which subsided ; but returned every ten or twenty minutes. The vesical pain had continued, but was mild when compared with the other. Dr. B. now ordered him a linament containing the extract of belladonna, warm hip bathing, mustard cataplasms to the inside of the thighs, and a full dose of morphine. He experienced no relief; therefore, it occurred to Dr. B. that the better plan would be to revulse on his bowels, with which view, Dr. B. prescribed for him pills of calomel and cathartic extract, and afterwards a solution of Epsom salt in an infusion of senna. Suffice it to say, that his symptoms soon yielded to this plan of treatment.

Dr. Bushe relates several other instances of a similar description. In one, the warm hip-bath, soda, and blue-pill with cathartic extract, removed the complaint in about three weeks. In another, the attacks, which were occasional, could always be prevented by an emollient injection, succeeded by a warm hip-bath, and an enema of starch and laudanum. In another case, in which there was retention of urine, the sphincter ani was so contracted that Dr. Bushe was obliged to divide it, before he could puncture the bladder.

On the whole, it must be evident, that Mr. Syme's unconditional opinion on the necessity of dividing the sphincter in the case of spasmodic contraction is too strong. We would say that in each case the cause should be investigated, and if possible, removed. If that is not possible, or if its removal is not sufficient for the cure of the complaint, then the sphincter will require division. But it would be subjecting some patients to a disagreeable operation unnecessarily, to make it a rule to divide the sphincter in all instances.

### X. Ulceration of the Rectum.

This forms the subject of the twelth chapter of Dr. Bushe's work, but is not treated of in Mr. Syme's.

In this chapter, Dr. Bushe confines himself to the ulceration of the rectum, arising from inflammation, and to that dependent on entanglement of fæces in the lacunæ. In subsequent chapters he treats of ulceration produced by the venereal poison and by malignant deposits.

In this, says Dr. Bushe, as in similar affections of other organs, there are three circumstances to be considered: firstly, the influence of the cause which produces it; secondly, the state of the constitution; and, thirdly, the situation it occupies. Thus, when it arises from the entanglement of fæces in the lacunæ, it is of moderate size, somewhat circular, deep, and surmounted by an indurated brim. In a sound constitution, though sometimes pretty extensive, it is generally superficial and without induration. In unhealthy persons it becomes inactive, and sometimes phagedenic. Finally, when within the limits of the sphincters, it is exquisitely painful, and the system sympathises as in fissure.

The symptoms of this disease, he continues, are pain, a sense of weight in the sacral region extending to the loins, vesical irritation, tenesmus, the discharge of a thin bloody fetid pus from the anus, besmearing of the fæces with the same, smarting, and even acute pain in the rectum, invariably increased by defecation ; and, when the ulceration extends low down, spasm of the sphincters with the other concomitants mentioned under the head of fissure.

If the finger be introduced, the ulcerated surface may be detected by its roughness ; but the better plan is to dilate the anus with the speculum, when the situation, extent, form and character of the disease can be easily determined. In many cases, however, nothing more will be necessary than to separate the buttocks, and press apart the edges of the anus with the fingers.

Mr. Colles recommends a blunt polished gorget, with its concavity looking towards the seat of the disease, to be passed upon the finger into the rectum ; then by everting the anus as much as we can, we shall obtain a full view of the ulcer by the light reflected from the gorget.

Dr. Bushe points out succinctly the reasons why ulceration of the rectum is difficult to heal : firstly, because, from the absence of valves in the portal system, and the depending situation of the hæmorrhoidal veins, they are loaded with blood, a condition which is still further increased by the accumulation of fæces in the lower bowels and the action of the sphincters; secondly, because the passage of the fæces contuses, and stretches the ulcerated surface ; thirdly, because, if the ulceration be within the limits of the sphincter, it is not only unduly compressed, but puckered ; fourthly, because the plicated condition of the mucous membrane, and the action of the sphincters, prevent the proper adjustment of suitable applications ; and fifthly, because we are unable to make pressure, a most efficient remedy in similar diseases of other parts.

Here we must stop. We think our readers will agree with us that Dr. Bushe's work contains much useful information. Of Mr. Syme's we can yet form no opinion. The limited range of subjects embraced by it has not admitted of our doing more than notice his observations on one particular disorder—spasmodic contraction of the sphincter ani.

In our next we shall return to our two authors, and to the diseases of the rectum. We trust that we shall increase our readers' acquaintance with both.

